# Type and intensity distribution of structured and incidental lifestyle physical activity of students and office workers: a retrospective content analysis

**DOI:** 10.1186/s12889-022-12999-z

**Published:** 2022-04-01

**Authors:** Birgit Wallmann-Sperlich, Peter Düking, Miriam Müller, Ingo Froböse, Billy Sperlich

**Affiliations:** 1 Institute of Sports Science, Julius-Maximilian University Würzburg, Judenbühlweg 11, 97082 Würzburg, Germany; 2 Integrative and Experimental Exercise Science and Training, Institute of Sport Science, University of Würzburg, Judenbühlweg 11, 97082 Würzburg, Germany; 3 Institute of Movement-Therapy and movement-oriented Prevention and Rehabilitation, German Sport University Cologne, Am Sportpark Müngersdorf 6, 50933 Köln, Germany

**Keywords:** Incidental lifestyle physical activity, Vigorous intermittent lifestyle physical activities, Physical activity, Diary, Structured physical activity, Context

## Abstract

**Background:**

Physical activity (PA) guidelines acknowledge the health benefits of regular moderate-to-vigorous physical activity (MVPA) regardless of bout duration. However, little knowledge exists concerning the type and intensity distribution of structured and incidental lifestyle PA of students and office workers. The present study aimed to i) assess the duration and distribution of intensity of MVPAs during waking hours ≥50% of heart rate reserve (HRR), ii) to identify the type of PA through diary assessment, iii) to assign these activities into structured and lifestyle incidental PA, and iv) to compare this information between students and office workers.

**Methods:**

Twenty-three healthy participants (11 students, 12 office workers) recorded heart rate (HR) with a wrist-worn HR monitor (Polar M600) and filled out a PA diary throughout seven consecutive days (i.e. ≥ 8 waking h/day). Relative HR zones were calculated, and PA diary information was coded using the Compendium of PA. We matched HR data with the reported PA and identified PA bouts during waking time ≥ 50% HRR concerning duration, HRR zone, type of PA, and assigned each activity to incidental and structured PA. Descriptive measures for time spend in different HRR zones and differences between students and office workers were calculated.

**Results:**

In total, we analyzed 276.894 s (76 h 54 min 54 s) of waking time in HRR zones ≥50% and identified 169 different types of PA. The participants spend 31.9 ± 27.1 min/day or 3.9 ± 3.2% of their waking time in zones of ≥50% HRR with no difference between students and office workers (*p* > 0.01). The proportion of assigned incidental lifestyle PA was 76.9 ± 22.5%.

**Conclusions:**

The present study provides initial insights regarding the type, amount, and distribution of intensity of structured and incidental lifestyle PA ≥ 50% HRR. Findings show a substantial amount of incidental lifestyle PA during waking hours and display the importance of promoting a physically active lifestyle. Future research could employ ambulatory assessments with integrated electronic diaries to detect information on the type and context of MVPA during the day.

**Supplementary Information:**

The online version contains supplementary material available at 10.1186/s12889-022-12999-z.

## Background

Physical inactivity is a global challenge [1] and facilitates the development of a variety of unfavorable health consequences such as non-communicable [2] or mental diseases [3]. To tackle the risk of physical inactivity, the World Health Organization (WHO) [4, 5] as well as many nations [6–8] provide physical activity (PA) recommendations to guide and inform governing bodies and individuals about the contribution of PA for promoting health and well-being across the life span [4]. The key message of the recently updated WHO guidelines on PA and sedentary behavior states that “every move counts”, emphasizing that i) the minimum weekly threshold of 150–300 min of moderate-to-vigorous-intensity physical activity (MVPA) as well as ii) the interruption of sedentary behavior with all kinds of PA (regardless of their intensity) essentially counteracts negative health outcomes [4].

Compared to the WHO PA guidelines of 2010, the updated guidelines do not recommend the accumulation of PA in at least 10 min bouts [9]. This recent modification reflects the growing evidence that PA of any bout duration is associated with improved health outcomes, including all-cause mortality [10, 11]. Additionally, the updated British PA guidelines explicitly acknowledge the health benefits of shorter exercise durations comprising of very vigorous-intensity PA such as sprinting or stair climbing [12] or performed as a high-intensity interval exercise (HIIT) with vigorous-intensity exercise “snacks” as described previously [13, 14]. The recognition of short (intense) PA benefiting various dimensions of health supports public health messages advocating lifestyle PA that are unlikely to last 10 min or longer, e.g. climbing staircases, carrying heavy shopping items or toddlers, managing housework [10].

.The aforementioned activities are categorized as incidental lifestyle PAs, i.e. activities as part of daily living and not intended for recreational or health purposes without requiring optional time [15]. Incidental PA represents the opposite of structured PA or exercise characterized by scheduled, pre-planned, and intentionally directed activities e.g. visiting a gym, jogging, cycling, or other activities for recreation, improving or maintaining physical fitness, performance, or health [16]. Incidental lifestyle PAs with an intensity exceeding 6 MET or ≥ 14 on Borg’s 6-to-20 scale [17] and shorter than < 5 min are defined as “vigorous intermittent lifestyle physical activities” [18]. However, little knowledge exists of (i) how frequently, (ii) with which type of behavior, (iii) in which PA domain (work, household, transport, or leisure), and (iv) to which extend incidental lifestyle PA, in general (long and shorter bouts), are distributed throughout the waking hours of different populations. To gather first information about the type and intensity distribution of incidental lifestyle PA, the selection of homogenous populations concerning their learning or working environment seems reasonable. For this study students and office-workers were selected, as these population groups are often prone to an inactive lifestyle [19–21].

Assessment of incidental lifestyle PAs or PAs of short duration is challenging since PA questionnaires are ineffective in capturing short and intermittent PA bouts and are prone to recall bias [22]. Advancements in wearable accelerometer-based technologies provide opportunities to reveal incidental PA throughout waking hours, however, accelerometry per se monitors a selection of external load markers and does not allow to judge internal loading. Additionally, obtaining valid accelerometer-based activity data is challenging because of correct accelerometer placement, data smoothing process, cut-off points, etc. [18].

Newly developed optical sensors now allow the continuous recording of heart rate (HR) [23–25] which probably is the most evaluated internal marker in various populations and settings in the field of exercise physiology for the assessment of cardiorespiratory load during movement of any kind including PA [22]. Unfortunately, current consumer-grade wearables do not automatically allow information regarding the type or domain of specific incidental PA behavior, for example, whether a specific HR response occurs due to stair climbing, vacuuming cleaning, carrying groceries, rushing to catch the bus, etc. Matching the HR response with subjective information about the specific type of PA such as through PA diaries would assist to understand the relative internal cardiorespiratory loading of certain (incidental lifestyle) PA. Information about the level of internal load during everyday PA behavior, as well as the frequency, duration, and specific type of PA, and how incidental lifestyle PA and structured PA relate to each other would deepen our knowledge in incidental lifestyle PA research. Deeper insights into incidental lifestyle PA would allow directing future public health messages advocating PA lifestyle behaviors, as incidental lifestyle PA does not encounter the multiple barriers to structured exercises, such as lack of time, costs, equipment, lack of skills, or poor fitness [15].

The present investigation aimed to i) assess the amount and distribution of intensity and duration of PAs during waking hours outreaching ≥50% of heart rate reserve (HRR) as an approximate of moderate-intensity PA > 3 MET, ii) to identify the type of these activities through diary assessment, iii) to assign these activities into structured PA and incidental lifestyle PA, and iv) to compare this information between two selected sample groups of students and office workers.

## Methods

### Study design

The observational study of students and office workers employed a mixed-method approach to assess the amount and distribution of intensity and duration of PAs during waking hours. All participants continuously were equipped with a wrist-worn HR monitor throughout the day for seven consecutive days (besides for charging) and recorded HR. The wrist-worn HR monitor was employed following the manufacturer’s recommendations (e.g. wearing location, settings, etc.). Furthermore, all participants were instructed to fill out a diary indicating their performed PA every 15 min throughout the day for 7 days. The study was approved by the ethical committee of the Sports Science Institute of the University of Würzburg (04/2021) and followed the Declaration of Helsinki [26].

### Participants

We recruited 23 healthy voluntary participants (11 university students (age range 18–23), 12 office workers (mean age 48 ± 7 yrs., 6 men)) as a convenient sample. We did not assess the sex and precise age of the students due to the anonymization of the rather small sample. All were informed about each experimental procedure and provided written consent to participate.

### Experimental procedures

#### Heart rate monitoring

A wrist-worn HR monitor (Polar M600, Polar Electro Oy, Kempele, Finland) with optical sensors and a sampling rate of 1 Hz recorded the HR throughout the day. This device provides accurate HR readings during periods of steady-state cycling, walking, jogging, and running and is most likely independent of sex, body mass index, maximal oxygen consumption, skin type, or wrist size [27].

#### Physical activity diary

In the present study, we employed a modified version of the Bouchard activity diary aiming to assess the type of PA subjectively instead of evaluating the energy expenditure [28]. Therefore, we only used the instrument’s grid-type table, which divides a day into 96 15-min periods over a 24-h period. We did not employ the original front page in our study (table of activities, energy cost, and corresponding categorical values) [28]). In contrast to Bouchard’s original diary, our participants were asked to record the i) type of activity into the grid table and ii) whenever they did not wear the smartwatch, instead of the code for each category of PA. All participants were asked to fill in the modified diary for seven consecutive days.

#### Data extraction and processing

All HR data were downloaded from each participant’s Polar Flow Applications (Polar, Polar Electro Oy, Kempele, Finland) as a Microsoft Excel file. The HR was smoothed using the average HR of each 15 s interval. Each participant’s maximum HR was estimated employing the equation “220 - age”, and resting HR was defined as the lowest, constant HR for 10 min recorded during the night while sleeping and averaged for 3 days. The traditional age equation formula (220-age) allows us to sufficiently approximate the HRmax in this rather young sample [29]. However, the formula may tend to underestimate HRmax in older populations with the effect of underestimating the true level of cardiorespiratory stress [29]. For the scope of this study, the underestimation of cardiorespiratory stress vs. overestimating cardiorespiratory stress seems to be a minor challenge.

Based on the individual’s maximal and resting HR, 10 individual relative HR zones were defined based on the individual’s heart rate reserve (HRR) which was subsequently divided into 10 equal 10% HRR zones. The HRR was calculated by subtracting the resting HR from the maximal HR [22].

To transfer each individual recorded HR into the individual relative HR for each value, the individual resting HR was subtracted from the smoothed average HR, then divided by the HRR and multiplied by 100. Time in each HRR-zone per day was calculated for each individual.

For the PA diary assessment, activities were coded and grouped using the five-digit code of the Compendium of Physical activities [30, 31]. If a participant reported a PA that was not listed in the compendium, a new code was created for this specific activity according to the coding scheme of the compendium (see suppl. 1).

All HR and subjective data were synchronized via timestamps obtained from the wrist-worn HR monitor and PA diary.

For data processing, we included all days with a waking time of ≥8 h/day. We defined waking time by i) wearing the wrist-worn heart rate monitor and ii) excluding time frames of ‘sleeping’ and ‘snoozing’ documented in the PA diary. For the scope of the data analysis, only moderate-to-vigorous PA were relevant. Thereby the HRR zones are normally classified as follows [22, 32]: 40- < 60% as moderate, 60- < 85% as vigorous (hard), 85 - < 100 as vigorous (very hard), and the HRR zone of 100% as maximal. In order not to overestimate moderate-to-vigorous PA, we only included PA with ≥50% of the HRR and parceled the data into 10% HRR zones to be more accurate through smaller zones (i.e. Zone 50–60% HRR, Zone 60–70% HRR, etc.). After identifying all PA bouts during waking time with an intensity ≥50% HRR, we determined the start and stop time, calculated the seconds in each zone through the objective data, and matched the HR-data with the reported specific type of PA from the diary. Then we listed the duration as well as the HRR zone. In case a participant entered two or more PA in the same 15-min period in the PA diary, then the values were assigned to the more vigorous PA. Values ≥50% HRR, which could not be assigned to any PA because of missing subjective PA diary information, were documented as “no answer”.

After classification of the PA type based on the information of the PA diary, we assigned the PA type to incidental or structured PA. Structured PA was defined as i) exercise or performing sports, ii) all activities in the categories conditioning exercise, running and sports, iii) for the activities mountain biking, dance workout/dance, aerobic, dancing, nordic walking, swimming. All other activities were assigned to incidental PA. For every specific type of PA ≥ 50%HRR, we listed the number of participants reporting the PA, the total duration during waking time, the frequency of the type of PA, and the mean duration of PA when occurring.

We calculated the mean minutes per day in zones ≥50% HRR. The percentage of waking time in zones ≥50% HRR was calculated by dividing the accumulated time in each zone and in the Zone ≥50% HRR through the total waking time. To identify the percentage of incidental PA compared to structured PA in zones ≥50% HRR, we divided the time of incidental PA through the total time in each zone only for the participants who featured PA bouts in this zone.

### Statistical analysis

Mean, standard deviation, median, and confidence intervals for time spend in different HRR zones was calculated. Most of the dependent variables (daily time in zone 50–100% HRR; percentage of waking time per day in Zone 50–100% HRR [%]; percentage of incidental PA compared to structured PA in Zone > 50% HRR [%]) were not normally distributed (Kolmogorov-Smirnov-Test). To explore differences between students and office workers in the zones of 50–100% of HRR we used the non-parametric Mann-Whitney-U-test. To prevent inflation of type 1 error, we applied an alpha level of *p* < 0.01. All statistical analysis were performed in the SPSS 23.0 (IBM Corp., Armonk, NY, USA) software package for Microsoft, and figures of descriptive numbers were prepared in Excel 2016.

## Results

In total, we recorded 179 days of 23 participants which resulted in 141 valid days (≥ 8 h of waking time) and 6.13 ± 1.01 valid days per participant. In total, we matched 6.951.254 s waking time (i.e. 49,114 ± 4334 s of waking wearing time per participant per valid day (13 h 38 min 34 s ± 1 h 12 min 23 s)) of objective HR with PA diary data and allocated the HR to the different HRR zones. In summary, 276.894 s (76 h 54 min 54 s) of waking time were in HRR zones ≥50% and were employed for further analyses including total time spend in different HRR zones, type of PA (169 different types), frequency of PA ≥ 50% HRR (total of 6.074 events), bout duration and allocation to structured or incidental PA (see Table 1).

**Table 1:** Descriptive measures of sample and analyzed wearing time (Mean ± SD)

	Total	Students	Office workers
Number of participants	23	11	12
Number of recorded days	179	85	94
Number of valid days (> 8 h of waking time)	141	62	79
Number of mean valid days / participant	6.13 (± 1.01)	5.64 (± 0.81)	6.58 (± 1.00)
Total recorded wearing time [s]	11.438.201 (18w 6d 9 h 16 min 41 s)	5.105.611 (8w 3d 2 h 13 min 31 s)	6.332.590 (10w 3d 7 h 3 min 10s)
Total duration of waking wearing time [s]	6.951.254 (11w 3d 10 h 54 min 1 s)	2.916.268 (4w 5d 18 h 4 min 28 s)	4.034.986 (6w 4d 16 h 49 min 46 s)
Mean total duration of waking wearing time / participant [s]	302.228 (± 60.311) (83 h 57 min 8 s)	265.115 (± 50.398) (73 h 38 min 35 s)	336.249 (± 48.227) (93 h 24 min 9 s)
Mean total duration of waking wearing time / participant / valid day [s]	49.114 (± 4334) (13 h 38 min 34 s)	46.847 (± 5152) (13 h 0 min 47 s)	51.191 (± 1895) (14 h 13 min 11 s)
Total duration of waking time in HRR zones ≥50% [s]	276.894 (76 h 54 min 54 s)	83.929 (23 h 18 min 49 s)	192.965 (53 h 36 min 5 s)
Mean total duration of waking time in HRR zones ≥50% per participant [s]	12.039 (± 11.576) (3 h 20 min 39 s)	7.630 (± 4.821) (2 h 7 min 10s)	16.080 (± 14.478) (4 h 28 min 0 s)
Mean total duration of waking time in HRR zones ≥50% / participant per valid day [s]	1912 (± 1626) (31 min 52 s)	1370 (± 843) (22 min 50s)	2408 (± 2020) (40 min 8 s)
Total frequency of bouts in waking time in HRR zones ≥50%	6.074	1.913	4.161
Daily mean total frequency of bouts in waking time in HRR zones ≥50% / participant (± SD)	264 (± 237)	174 (± 100)	347 (± 296)
Daily mean total frequency of bouts in waking time in HRR zones ≥50% / participant / valid day (± SD)	42 (± 34)	31 (± 18)	53 (± 41)
Number of types of PA reported by all participants in HRR zones ≥50%	169	82	112

**Fig. 1:**
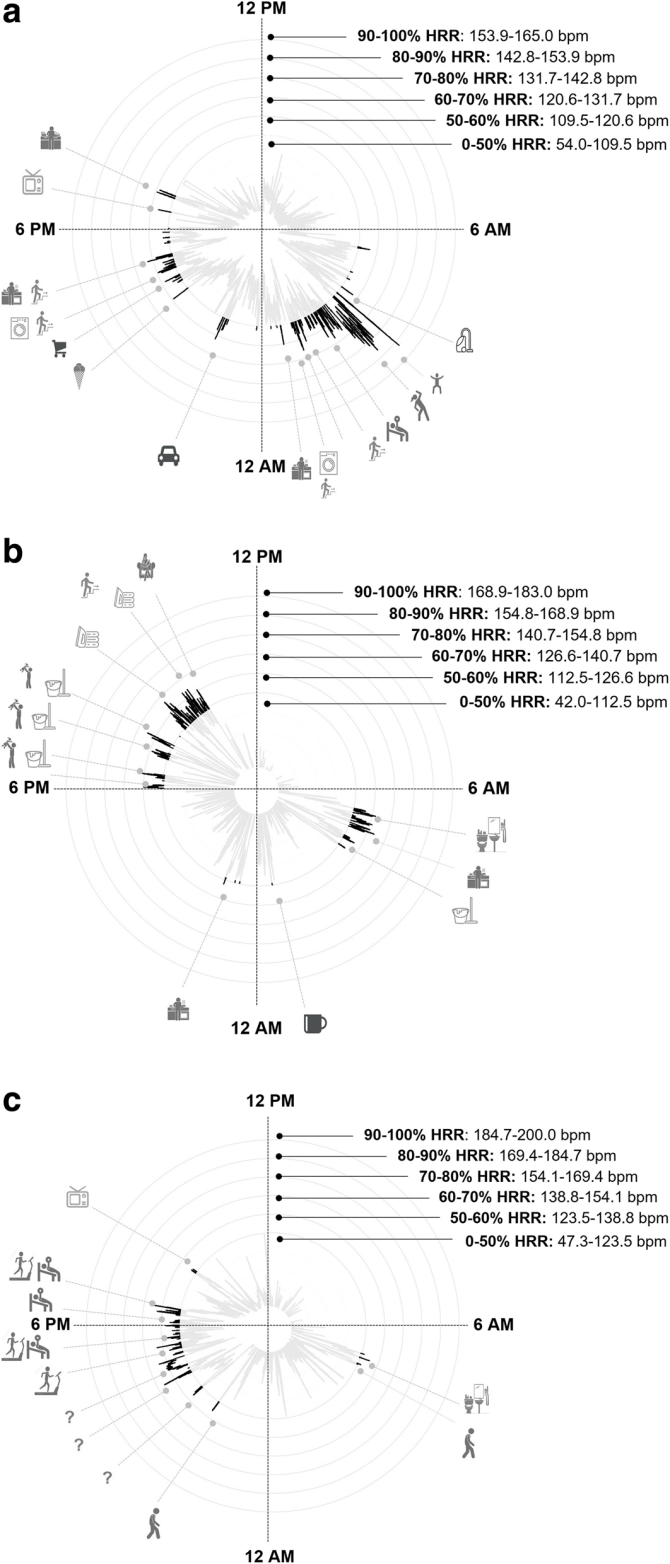
Examples of 24-h heart rate patterns of **a**) an office worker with a day off, **b** an office worker in home office and **c**) an university student with matched physical activity information from the physical activity diary. Heart rate zones reflect the relative zones of the individual heart rate reserve

Examples of individual HR patterns with matched PA information of participants are illustrated in Fig. 1a-c.

The participants spend 31.9 ± 27.1 min/day or 3.9 ± 3.2% of their waking time in zones of ≥50% HRR with no difference between students and office workers (*p* > 0.01). Office workers spent more time in HRR zones ≥90% compared to students (1.73 ± 2.54 min/day vs. 0.03 ± 0.61 min/day; *p* < .01; 0.20 ± 0.29% vs. 0.003 ± 0.007%, *p* < .01) (see Table 2).

**Table 2:** Descriptive measures (mean ± standard deviation, median; 95% confidence interval) and the difference between students and office workers concerning time per day of activities ≥50% heart rate reserve, the percentage of time compared to total waking wearing time in heart rate reserve zones ≥50%, and the percentage of assigned incidental lifestyle physical activity compared to assigned structured PA in heart rate reserve zones ≥50%

	All (*n* = 23)	Students (*n* = 11)	Workers (*n* = 12)	Z *p*
Daily time in Zone 50–100% HRR [min]	31.86 ± 27.1 27.1 [20.14; 43.58]	22.84 ± 14.05 23 [13.40; 32.38]	40.13 ± 33.66 28.36 [18.74; 61.51]	−.92 .38
Daily time in Zone 50–60% HRR [min]	21.87 ± 17.15 15.57 [14.45; 29.28]	16.85 ± 10.93 15.57 [9.51; 24.19]	26.47 ± 20.76 16.47 [13.28; 39.66]	−.86 .41
Daily time in Zone 60–70% HRR [min]	5.08 ± 5.21 4.58 [2.82; 7.33]	3.80 ± 2.95 4.58 [1.82; 5.78]	6.24 ± 6.58 4.66 [2.07; 10.42]	−.86 .41
Daily time in Zone 70–80% HRR [min]	2.79 ± 5.71 0.78 [0.32; 5.26]	1.73 ± 2.17 0.74 [0.28; 3.19]	3.77 ± 7.67 1.25 [− 1.10; 8.64]	−.59 .57
Daily time in Zone 80–90% HRR [min]	1.21 ± 1.97 0.4 [0.35; 2.06]	0.43 ± 0.90 0 [− 0.17; 1.04]	1.91 ± 2.43 1.04 [0.37; 3.46]	−2.2 .03
Daily time in Zone 90–100% HRR [min]	0.92 ± 2.0 0 [0.05; 1.78]	0.03 ± 0.61 0 [− 0.02; 0.07]	1.73 ± 2.54 0.22 [0.12; 3.35]	−2.69 .01*
Percentage of waking time per day in Zone 50–100% HRR [%]	3.87 ± 3.22 3.34 [2.48; 5.26]	2.91 ± 1.71 3.33 [1.76; 4.06]	4.74 ± 4.03 3.34 [2.18; 7.31]	−.62 .57
Percentage of time per day in Zone 50–60% HRR [%]	2.67 ± 2.05 1.94 [1.78; 3.56]	2.17 ± 1.36 1.94 [1.25; 3.08]	3.13 ± 2.5 1.98 [1.54; 4.72]	−.55 .61
Percentage of time per day in Zone 60–70% HRR [%]	0.62 ± 0.62 0.58 [0.35; 0.89]	0.48 ± 0.36 .58 [0.24; 0.72]	0.74 ± 0.79 0.55 [0.24; 1.24]	−.68 .53
Percentage of time per day in Zone 70–80% HRR [%]	0.33 ± 0.68 0.11 [0.04; 0.63]	0.21 ± 0.25 .09 [0.04; 0.38]	0.45 ± 0.91 0.15 [− 0.13; 1.02]	−.59 .57
Percentage of time per day in Zone 80–90% HRR [%]	0.14 ± 0.23 0.05 [0.04;0.24]	0.05 ± 0.11 0 [− 0.02; 0.12]	0.23 ± 0.29 0.12 [0.04; 0.41]	−2.2 .03
Percentage of time per day in Zone 90–100% HRR [%]	0.11 ± 0.23 0 [0.01; 0.21]	0.003 ± 0.007 0 [− 0.002; 0.01]	0.20 ± 0.29 0.03 [0.02; 0.39]	−2.69 .01*
Percentage of incidental PA compared to structured PA in Zone ≥50% HRR [%]	76.89 ± 22.51 (*n* = 23) 77.37 [67.15; 86.62]	70.27 ± 24.18 (*n* = 11) 73.15 [54.02; 86.51]	82.96 ± 19.96 (*n* = 12) 93.50 [70.28; 95.64]	− 1.51 .15
Percentage of incidental PA compared to structured PA in Zone 50–60% HRR [%]	87.53 ± 16.58 (*n* = 23) 94.26 [80.36; 94.70]	77.59 ± 19.44 (*n* = 11) 76.93 [64.51; 90.63]	96.66 ± 4.0 (*n* = 12) 98.33 [94.12; 99.20]	−2.39 .02
Percentage of incidental PA compared to structured PA in Zone 60–70% HRR [%]	70.50 ± 29.90 (*n* = 23) 74.11 [57.57; 83.43]	60.12 ± 33.15 (*n* = 11) 67.11 [37.85; 82.39]	80.01 ± 24.14 (*n* = 12) 87.87 [64.68; 95.35]	− 1.52 .15
Percentage of incidental PA compared to structured PA in Zone 70–80% HRR [%]	47.88 ± 44.78 (*n* = 19) 29.76 [26.30; 69.47]	43.17 ± 43.82 (*n* = 8) 27.91 [6.54; 79.80]	51.31 ± 47.27 (*n* = 11) 30.21 [19.55; 83.07]	−.68 .55
Percentage of incidental PA compared to structured PA in Zone 80–90% HRR [%]	36.29 ± 47.12 (*n* = 15) 4.44 [10.20; 62.38]	20.00 ± 44.72 (*n* = 5) 0 [− 35.53; 75.53]	44.43 ± 48.41 (*n* = 10) 19.95 [9.80; 79.07]	− 1.45 .21
Percentage of incidental PA compared to structured PA in Zone 90–100% HRR [%]	30.32 ± 45.70 (*n* = 11) 0 [− 0.38; 61.03]	0 ± 0 (*n* = 2) 0	37.07 ± 48.27 (*n* = 9) 1.97 [− 0.04; 74.17]	−1.30 .33

*(*p* ≤ 0.01)

Comparing the proportions of assigned incidental and assigned structured PA ≥ 50% HRR, 76.9 ± 22.5% was spent in incidental PA with no differences between students and office workers. In zone 90–100% of HRR, the proportion of assigned incidental PA compared to assigned structured PA was 30.3 ± 45.7%.

Tables 3 and 4 summarize the reported type of PA (incidental and structured) accomplished by all students and office workers (all types of physical activities are reported in the supplementary material). Within the incidental PA of students’ leisure time, activities such as self-care and miscellaneous activities (total of 403.6 min) and transportation activities (total of 306.5 min) were most often reported. Office workers reported most often incidental PA in the categories transportation (total of 960.5 min), household activities (total of 652.6 min), and leisure time activities (total of 573.6 min) in HRR zones ≥50%.

**Table 3:** Description of reported incidental lifestyle PA and structured PA of *n* = 11 students in the HRR zone ≥50% with total reported duration during waking time, frequency, and mean duration of PA when it occurred (total duration of waking time in HRR zones ≥50% = 83.929 s (23.31 h); total frequency in waking time in HRR zones ≥50% = 1913)

Domain	Major heading of Compendium	Total duration in activity in waking time ([s]	Frequency of activity in waking time	Mean duration of activity [s]
Household		4.000	102	42.59
	Home activities	3.643	88	45.69
	Home repair	357	14	22.43
Leisure Time		24.216	595	38.78
	Inactivity quiet/light	3145	92	50.00
	Miscellaneous	14.750	351	36.20
	Playing music	941	23	32.92
	Self-care	5.168	120	41.33
	Sports	212	9	17.06
Occupation/ Study		4.401	91	47.17
	Occupation/student activities	3.833	76	49.63
	Volunteer activities	568	15	32.42
Transportation		18.387	398	39.04
	Combined transportation	1.725	36	53.01
	Bicycling	982	27	28.97
	Transportation	7.995	170	39.93
	Walking	7.685	165	30.41
Others/Combination		2.856	57	34.94
	Home activities; walking	927	12	53.15
	Inactivity quiet/light; self-care	637	14	32.91
	Inactivity quiet/light; self-care; walking	8	1	8.00
	Self-care; walking	1.284	30	34.26
Not Specified		1.979	45	33.74
Sport		28.090	625	41.48
	Conditioning exercise	5.828	141	33.40
	Dancing	99	5	19.80
	Running	9.097	174	53.76
	Sports	10.745	267	35.15
	Walking	1.931	28	57.30
	Water activities	390	10	37.22

**Table 4:** Description of reported incidental lifestyle PA and structured PA of *n* = 12 office workers in the HRR zone ≥50% with total reported duration during waking time, frequency, and mean duration of PA when it occurred (total duration of waking time in HRR zones ≥50% = 192.965 s (53.60 h); total frequency in waking time in HRR zones ≥50% = 4161)

Domain	Major heading of Compendium	Total duration in activity in waking time [s]	Frequency of activity in waking time	Mean duration activity (s)
Household		39.155	837	36.42
	Home activities	36.217	777	36.47
	Home repair	2.377	44	44.98
	Lawn and Garden	561	16	29.71
Leisure Time		34.416	837	39.77
	Inactivity quiet/light	10.036	243	40.37
	Miscellaneous	896	27	25.86
	Self-care	23.484	567	43.62
Occupation/ Study		8.830	240	32.61
	Occupation/student activities	8.830	240	32.61
Transportation		57.628	1.367	49.77
	Combined transportation	1.167	23	78.61
	Bicycling	5.872	154	39.32
	Transportation	10.064	241	47.15
	Walking	40.525	949	46.67
Others/Combinations		7.753	161	56.89
	Conditioning exercise; self-care	101	5	20.20
	Home activities; walking	2.979	40	118.92
	Home activities; self-care	1.384	31	36.58
	Home activities; transportation	1.235	30	27.00
	Home activities; transportation; walking	1.598	37	39.17
	Inactivity quiet/light; self-care	115	6	27.10
	Miscellaneous; self-care	22	2	11.00
	Occupation/student activities; walking	258	7	27.08
	Self-care; walking	61	3	17.00
Not Specified		118	4	29.50
Sport		45.065	715	47.05
	Bicycling	9.780	210	47.92
	Conditioning exercise	32.438	428	56.04
	Dancing	2.772	68	38.18
	Sports	75	9	8.30

## Discussion

The main findings of our study are that our sample achieved about 30 min per day in zones ≥50% HRR and that more than 75% of the PA in ≥50% HRR zones were accomplished through incidental lifestyle PA, with little difference between students and office workers. We furthermore identified that most of the incidental lifestyle PA ≥ 50% HRR in students included leisure and transportation activities and in office workers transportation, household, and leisure time activities.

Since the mean duration of PA ≥ 50% HRR was > 30 min per day in most participants, our sample sufficiently achieved the updated WHO recommendation for PA and sedentary behavior [9]. Considering the predominant short duration of the bouts in zones ≥50% HRR it becomes obvious that most of the activities in ≥50% HRR zones lasted less than 10 min. Recent evidence, however, supports the fact that PA of any bout duration is associated with improved health outcomes [10, 11]. Most of the accomplished PA ≥ 50% HRR in the present study (21.87 ± 17.15 min/day) comprised of moderate-intensity PA (i.e. 50 - < 60% HRR) [22, 33]. However, almost one-third of the PA (10.0 ± 12.1 min/day) included vigorous, very vigorous, or maximal intensities (≥ 60% HRR) [22, 33]. Following epidemiological evidence, a proportion of ≥30% vigorous PA of total PA suggests additional health benefits compared to an equivalent amount of moderate-intensity PA [34–36]. As stated previously [23], future studies should further investigate and compare the roles of low to vigorous-intensity activities (independent of quantifying total activity energy expenditure) for health promotion, disease prevention, and management, so that public health messaging can be directed more specifically to the type and proportion of different intensity PA should ideally have.

Interestingly, the proportion of assigned incidental compared to assigned structured PA was considerably high with more than 75% of overall PA in zones ≥50% HRR in the two studied sample populations. The high contribution of incidental PA in our results resembles in part the findings of a representative study in the population of US adults [37], in which lifestyle activities were more frequently reported than sports and/or recreational activities. Our findings reveal to some extent the moderate- and the vigorous-intensities of incidental lifestyle PA and consequently exhibit the importance of promoting a physically active lifestyle to achieve the minimum recommended PA for a healthy life. Especially in physically inactive populations reporting a perceived lack of time or a low priority for exercising [38], lifestyle embedded PA could play an essential role to engage in sufficient MVPA.

In respect to the high contribution of incidental PA in more vigorous intensity zones, the participants in this study displayed a considerable amount of vigorous lifestyle PA [15, 18] (i.e. Home activities (‘cleaning’, ‘cooking’, ‘putting away groceries’, ‘hanging laundry’, etc.), ‘sightseeing’, ‘celebrating’, ‘playing piano’, ‘biking’, ‘walking’, ‘climbing stairs’ etc.). Following the results of previous experimental studies, also short intense exercise bouts of incidental PA could, at least to some extent, positively impact cardiorespiratory fitness [13, 14]. To date, the knowledge about vigorous intermitted (short) lifestyle PAs is limited [18] and the relatively high contribution of these activities in our study sample supports the importance and need for the recently established research framework to better understand the health potential of vigorous intermitted lifestyle PA [18]. For example, it seems meaningful i) to better understand the contribution of vigorous intermittent lifestyle physical activities in PA patterns, ii) to recognize and understand the short and long-term dose-response of vigorous intermittent lifestyle PA concerning health, and iii) to gain knowledge about how to convince and empower people to be more physically active in their daily lives [15].

We detected a marginally greater contribution of time in the ≥90% HRR zone in office workers than in students (1.73 ± 2.54 min vs. 0.03 ± 0.61 min). Recognized PA of the diary was predominately identified during leisure time with activities such as ‘jogging’, ‘walking the dog’, ‘mountain biking’, ‘circuit training’, ‘treadmill’, ‘Qi Gong (shaking exercise)’ etc. but also unstructured activities such as ‘getting changed’ in office workers. One reason for little time spent in ≥90% HHR may also arise from the inert kinetics of HR. Typically, after the onset of vigorous activity, the neuro-humoral and metabolic mechanisms stimulating HR increase require several seconds to meet the oxygen need of the working muscle, and usually HR plateaus after approximately 60s with high intensity [39]. Thus, any type of vigorous activity < 60 s will not be sufficiently described by continuous HR recording.

In both samples, transportation activities such as walking, cycling, and transportation or combinations of these contribute considerably to the PA behaviors exceeding 50% HRR, which is in line with previous findings [40] and underpins the importance of promoting active transportation for health and PA promotion [41–43]. Household activities ≥50% HRR were more present in office workers than in students, potentially because office workers live in households with more than one person and take more actions for cleaning, washing, grocery shopping, etc. Students may often live in single-room flats, or in their family homes, where they might engage in fewer household activities. Therefore, cognitive restructuring [44, 45] of often unpopular household activities, i.e. highlighting the health potential of carrying heavy shopping bags or vacuum cleaning could be a strategy to support PA promotion and achieving recommended PA. Unexpectedly, leisure time self-care activities such as showering, eating, dressing (see supplementary material 1) often exceeded 50% HRR. This could be due to frequent changes in body positions during showering and dressing, possible time constraints or possible inaccuracy i) arising from faulty PA diary recording, or ii) HR monitoring due to fluid interference with the optical sensors of the wrist-worn HR monitor.

## Limitations

Assessing HR and linking the data to PA has some limitations as the HR not only responds to the oxygen needs in connection with PA. Numerous other factors including changes in body position (e.g. moving from supine to erect posture in healthy adults may induce an immediate increase in heart rate) [46], smoking [47], consumption of alcohol [48], and neurohormonal factors such as psychological stress [49] and emotional circumstances may cause sympathetic reactivity (e.g. due to scary or enjoyable situations) triggering plasma catecholamines increase, which is a main driver for HR elevation [50].

Hence, changes in HR throughout the day are the result of a quite complex interplay and may affect present HR data. However, controlling these factors while freely living is challenging. The HR monitor employed in the present study is accurate during periods of steady-state endurance exercise [27] but has not been validated for all-day activities. Furthermore, the validation of the used HRmax formula in our sample and further integration of anthropometric variables could add accuracy to future studies. Additionally, the sex of the student population was not recorded, which may have an impact on the interpretation of the results.

In some cases, reported PAs in the diary, e.g. sitting and working which were assigned to sedentary behaviors (i.e. a metabolic equivalent of < 1.5 MET [51]) revealed short durations with heart rates ≥50% HRR zones. These findings point out that within sedentary or low-intense behaviors (e.g, sitting, working, or driving a car) short durations of intense cardio-respiratory loading may occur. One explanation could be that these moderate-to-vigorous-intensity behaviors are not always captured by the relatively long diary reporting time frame (i.e. 15 min) for PA assessment [28]. Another reason could be that participants reported a (longer) bout of intense PA (e.g. jogging) within one or two 15 min-frames and this activity could have lasted several minutes (e.g. 3 min) within the proceeding 15-min frame but without mentioning in the PA diary. Another reason could be that the HR elevation may have been caused by non-movement situations, i.e. psychological stress.

The identified challenges suggest a methodological modification for future studies. One modification could be to ask participants to document non-movement events that may have triggered a rise in HR (i.e. emotional event, alcohol intake, smoking, etc.). A more sophisticated possibility could be to employ ambulatory assessment including movement monitoring (i.e. external load) via e.g. accelerometry and HR (internal load) with ecological momentary assessment, such as electronic diaries [52] to capture real-time self-reported information. The strengths of ambulatory assessments are the acquisition of data near real-time, thereby i) minimizing retrospective biases in real-world settings and ii) enabling ecological valid findings [52].

## Conclusion

The present study provides initial insights regarding the type, amount, and distribution of intensity of structured and incidental lifestyle PA ≥ 50% HRR during the day in students and office workers. The present findings revealed that more than 75% of the PA ≥ 50% HRR was assigned to incidental lifestyle PA and that a substantial amount was spent engaging in vigorous intensity. The present data underline the importance of promoting a physically active lifestyle next to structured PA and points to the need for future research to better understand the health potential of incidental lifestyle PA. Therefore ambulatory assessments with integrated electronic diaries could help to detect information on the type and context of MVPA during the day.

## Supplementary Information

**Additional file 1.**

## Data Availability

The datasets of the current study are available from the corresponding author on reasonable request.

## Abbreviations

PA: Physical activity
MVPA: Moderate-to-vigorous physical activity
HR: Heart rate
HRR: Heart rate reserve

## References

[1] Guthold R, Stevens GA, Riley LM, Bull FC (2018). Worldwide trends in insufficient physical activity from 2001 to 2016: a pooled analysis of 358 population-based surveys with 1.9 million participants. Lancet Glob Health.

[2] Lee IM, Shiroma EJ, Lobelo F, Puska P, Blair SN, Katzmarzyk PT (2012). Effect of physical inactivity on major non-communicable diseases worldwide: an analysis of burden of disease and life expectancy. Lancet..

[3] Schuch FB, Vancampfort D, Richards J, Rosenbaum S, Ward PB, Stubbs B (2016). Exercise as a treatment for depression: a meta-analysis adjusting for publication bias. J Psychiatr Res.

[4] Bull FC, Al-Ansari SS, Biddle S, Borodulin K, Buman MP, Cardon G (2020). World Health Organization 2020 Guidelines on physical activity and sedentary behaviour.

[5] World Health Organization W (2010). Global recommendations on physical activity for health.

[6] Rütten A, Pfeifer K (2016). Nationale Empfehlungen für Bewegung und Bewegungsförderung.

[7] Piercy KL, Troiano RP, Ballard RM, Carlson SA, Fulton JE, Galuska DA (2018). The physical activity guidelines for Americans. JAMA..

[8] Tremblay MS, Warburton DE, Janssen I, Paterson DH, Latimer AE, Rhodes RE (2011). New Canadian physical activity guidelines. Appl Physiol Nutr Metab..

[9] Bull FC, Al-Ansari SS, Biddle S, Borodulin K, Buman MP, Cardon G (2020). World Health Organization 2020 guidelines on physical activity and sedentary behaviour. Br J Sports Med.

[10] Jakicic JM, Kraus WE, Powell KE, Campbell WW, Janz KF, Troiano RP (2019). Association between bout duration of physical activity and health: systematic review. Med Sci Sports Exerc.

[11] Murphy MH, Lahart I, Carlin A, Murtagh E (2019). The effects of continuous compared to accumulated exercise on health: a Meta-analytic review. Sports Med.

[12] Gibson-Moore H (2019). UK chief medical officers’ physical activity guidelines 2019: What’s new and how can we get people more active?. Nutr Bull.

[13] Jenkins EM, Nairn LN, Skelly LE, Little JP, Gibala MJ (2019). Do stair climbing exercise "snacks" improve cardiorespiratory fitness?. Appl Physiol Nutr Metab.

[14] Little JP, Langley J, Lee M, Myette-Cote E, Jackson G, Durrer C (2019). Sprint exercise snacks: a novel approach to increase aerobic fitness. Eur J Appl Physiol.

[15] Stamatakis E, Johnson NA, Powell L, Hamer M, Rangul V, Holtermann A (2019). Short and sporadic bouts in the 2018 US physical activity guidelines: is high-intensity incidental physical activity the new HIIT?. Br J Sports Med.

[16] Caspersen CJ, Powell KE, Christenson GM (1985). Physical activity, exercise, and physical fitness: definitions and distinctions for health-related research. Public Health Rep.

[17] Borg GA (1982). Psychophysical bases of perceived exertion. Med Sci Sports Exerc.

[18] Stamatakis E, Huang BH, Maher C, Thøgersen-Ntoumani C, Stathi A, Dempsey PC (2021). Untapping the health enhancing potential of vigorous intermittent lifestyle physical activity (VILPA): rationale, scoping review, and a 4-pillar research framework. Sports Med.

[19] Castro O, Bennie J, Vergeer I, Bosselut G, Biddle SJH (2020). How sedentary are University students? A systematic review and Meta-analysis. Prev Sci.

[20] Prince SA, Elliott CG, Scott K, Visintini S, Reed JL (2019). Device-measured physical activity, sedentary behaviour and cardiometabolic health and fitness across occupational groups: a systematic review and meta-analysis. Int J Behav Nutr Phys Act.

[21] Wallmann-Sperlich B, Chau JY, Froboese I (2017). Self-reported actual and desired proportion of sitting, standing, walking and physically demanding tasks of office employees in the workplace setting: do they fit together?. BMC Res Notes.

[22] Strath SJ, Kaminsky LA, Ainsworth BE, Ekelund U, Freedson PS, Gary RA (2013). Guide to the assessment of physical activity: clinical and research applications: a scientific statement from the American Heart Association. Circulation..

[23] Ding D, Ramirez Varela A, Bauman AE, Ekelund U, Lee IM, Heath G (2020). Towards better evidence-informed global action: lessons learnt from the lancet series and recent developments in physical activity and public health. Br J Sports Med.

[24] Sallis JF, Saelens BE (2000). Assessment of physical activity by self-report: status, limitations, and future directions. Res Q Exerc Sport.

[25] Düking P, Giessing L, Frenkel MO, Koehler K, Holmberg HC, Sperlich B (2020). Wrist-worn Wearables for monitoring heart rate and energy expenditure while sitting or performing light-to-vigorous physical activity: validation study. JMIR mHealth uHealth.

[26] World Medical Association Declaration of Helsinki (2013). Ethical principles for medical research involving human subjects. JAMA..

[27] Horton JF, Stergiou P, Fung TS, Katz L (2017). Comparison of polar M600 optical heart rate and ECG heart rate during exercise. Med Sci Sports Exerc.

[28] Bouchard C, Tremblay A, Leblanc C, Lortie G, Savard R, Theriault G (1983). A method to assess energy expenditure in children and adults. Am J Clin Nutr.

[29] Tanaka H, Monahan KD, Seals DR (2001). Age-predicted maximal heart rate revisited. J Am Coll Cardiol.

[30] Ainsworth BE, Haskell WL, Leon AS, Jacobs DR, Montoye HJ, Sallis JF (1993). Compendium of physical activities: classification of energy costs of human physical activities. Med Sci Sports Exerc.

[31] Ainsworth BE, Haskell WL, Herrmann SD, Meckes N, Bassett DR, Tudor-Locke C (2011). Compendium of physical activities: a second update of codes and MET values. Med Sci Sports Exerc.

[32] Pescatello L, Arena R, Riebe D, Thompson P (2014). ACSM’s guidelines for testing and prescription.

[33] Physical Activity Guidelines Advisory Committee (2008). Physical Activity Guidelines Advisory Committee Report, 2008.

[34] Gebel K, Ding D, Chey T, Stamatakis E, Brown WJ, Bauman AE (2015). Effect of moderate to vigorous physical activity on all-cause mortality in middle-aged and older Australians. JAMA. Intern Med.

[35] Gebel K, Ding D, Bauman AE (2014). Volume and intensity of physical activity in a large population-based cohort of middle-aged and older Australians: prospective relationships with weight gain, and physical function. Prev Med.

[36] Rey Lopez JP, Gebel K, Chia D, Stamatakis E (2019). Associations of vigorous physical activity with all-cause, cardiovascular and cancer mortality among 64 913 adults. BMJ Open Sport Exerc Med.

[37] Welk GJ, Kim Y (2015). Context of physical activity in a representative sample of adults. Med Sci Sports Exerc.

[38] Hoare E, Stavreski B, Jennings GL, Kingwell BA (2017). Exploring motivation and barriers to physical activity among active and inactive Australian adults. Sports..

[39] Poole DC, Jones AM (2017). Measurement of the maximum oxygen uptake V̇o(2max): V̇o(2peak) is no longer acceptable. J Appl Physiol (1985).

[40] Buehler R, Pucher J, Merom D, Bauman A (2011). Active travel in Germany and the U.S. contributions of daily walking and cycling to physical activity. Am J Prev Med.

[41] Mueller N, Rojas-Rueda D, Cole-Hunter T, de Nazelle A, Dons E, Gerike R (2015). Health impact assessment of active transportation: a systematic review. Prev Med.

[42] Sallis JF, Frank LD, Saelens BE, Kraft MK (2004). Active transportation and physical activity: opportunities for collaboration on transportation and public health research. Transp Res A Policy Pract.

[43] Saunders LE, Green JM, Petticrew MP, Steinbach R, Roberts H (2013). What are the health benefits of active travel? A systematic review of trials and cohort studies. PLoS One.

[44] Foreyt JP (2005). Need for lifestyle intervention: how to begin. Am J Cardiol.

[45] Costain L, Croker H (2005). Helping individuals to help themselves. Proc Nutr Soc.

[46] Smith JJ, Porth CM, Erickson M (1994). Hemodynamic response to the upright posture. J Clin Pharmacol.

[47] Cryer PE, Haymond MW, Santiago JV, Shah SD (1976). Norepinephrine and epinephrine release and adrenergic mediation of smoking-associated hemodynamic and metabolic events. N Engl J Med.

[48] Spaak J, Merlocco AC, Soleas GJ, Tomlinson G, Morris BL, Picton P (2008). Dose-related effects of red wine and alcohol on hemodynamics, sympathetic nerve activity, and arterial diameter. Am J Physiol Heart Circ Physiol.

[49] Valentini M, Parati G (2009). Variables influencing heart rate. Prog Cardiovasc Dis.

[50] Carter JR, Ray CA (2009). Sympathetic neural responses to mental stress: responders, nonresponders and sex differences. Am J Physiol Heart Circ Physiol.

[51] Tremblay MS, Aubert S, Barnes JD, Saunders TJ, Carson V, Latimer-Cheung AE (2017). Sedentary behavior research network (SBRN) – terminology consensus project process and outcome. Int J Behav Nutr Phys Act.

[52] Reichert M, Giurgiu M, Koch E, Wieland LM, Lautenbach S, Neubauer AB (2020). Ambulatory assessment for physical activity research: state of the science, best practices and future directions. Psychol Sport Exerc.

