# Supplementary material for: Type and intensity distribution of structured and incidental lifestyle physical activity of students and office workers: a retrospective content analysis
Source: BMC Public Health. 2022 Apr 1;22:634. doi: 10.1186/s12889-022-12999-z (PMC8976323; doi:10.1186/s12889-022-12999-z)
Supplement: Supplementary file 1 — Additional file 1. [file 12889_2022_12999_MOESM1_ESM.docx]

**Supplementary**

Table 1: Description of the specific type of incidental lifestyle PA and the specific type of structured PA of n=11 students in the different heart rate reserve (HRR) zones, the number of participants per activity and their total duration during waking time, their frequency, and their mean duration of activity when it occurred (total duration of waking time in HRR zones ≥ 50% = 83929 s (23.31 h); total frequency in waking time in HRR zones ≥ 50% = 1913)

| **Domain** | **Major heading of**  **compendium** | **Code** | **Type of activity** | **Zone of HRR %** | **Number of participants mentioning the activity** | **Total duration in activity during waking time [s]** | **Frequency of activity in waking time** | **Mean duration of each activity [s]** |
| --- | --- | --- | --- | --- | --- | --- | --- | --- |
| **Incidental Lifestyle PA** | | | | | | | | |
| Household | Home Activities | | | | | | | |
|  |  | 05031 | cleaning | 50-60 | 5 | 846 | 26 | 32.5 |
|  |  |  |  | 60-70 | 1 | 14 | 1 | 14.0 |
|  |  | 05031; 05213 | cleaning; packing bag | 50-60 | 1 | 256 | 7 | 36.6 |
|  |  |  |  | 60-70 | 1 | 63 | 2 | 31.5 |
|  |  | 05041 | wash dishes | 50-60 | 1 | 35 | 2 | 17.5 |
|  |  | 05050 | cooking/food preparation | 50-60 | 1 | 338 | 7 | 48.3 |
|  |  |  |  | 60-70 | 1 | 15 | 1 | 15.0 |
|  |  | 05055 | putting away groceries | 50-60 | 1 | 293 | 5 | 58.6 |
|  |  | 05058 | market (old town) | 50-60 | 1 | 169 | 4 | 42.3 |
|  |  | 05060 | food shopping | 50-60 | 5 | 351 | 12 | 29.3 |
|  |  | 05091 | hanging laundry | 50-60 | 1 | 727 | 10 | 72.7 |
|  |  | 05213 | packing bag | 50-60 | 3 | 378 | 10 | 37.8 |
|  |  | 05214 | unpacking bag | 50-60 | 1 | 158 | 1 | 158.0 |
|  |  |  |  |  |  |  |  |  |
|  | Home Repair | | | | | | | |
|  |  | 06060 | set up furniture | 50-60 | 1 | 296 | 10 | 29.6 |
|  |  |  |  | 60-70 | 1 | 61 | 4 | 15.3 |
|  |  |  |  |  |  |  |  |  |
| Leisure Time | Inactivity quiet/light | | | | | | | |
|  |  | 07009 | getting up | 50-60 | 1 | 278 | 5 | 55.6 |
|  |  | 07011 | lying | 50-60 | 3 | 239 | 6 | 39.8 |
|  |  | 07012 | lying on bed/couch | 50-60 | 1 | 271 | 2 | 135.5 |
|  |  | 07013 | resting/chilling | 50-60 | 3 | 1075 | 37 | 29.1 |
|  |  |  |  | 60-70 | 2 | 157 | 4 | 39.3 |
|  |  | 07014 | waking up | 50-60 | 1 | 210 | 2 | 105.0 |
|  |  | 07015 | watching television, series, movies, Netflix, Youtube | 50-60 | 1 | 125 | 2 | 62.5 |
|  |  | 07015; 07017 | watching television, series, movies, Netflix, Youtube; mobile (e.g. Social Media) | 50-60 | 1 | 83 | 5 | 16.6 |
|  |  | 07021 | sitting | 50-60 | 5 | 665 | 27 | 24.6 |
|  |  |  |  | 60-70 | 1 | 25 | 1 | 25.0 |
|  |  | 07040 | standing | 50-60 | 1 | 17 | 1 | 17.0 |
|  |  |  |  |  |  |  |  |  |
|  | Miscellaneous | | | | | | | |
|  |  | 09000 | playing board game | 50-60 | 2 | 74 | 3 | 24.7 |
|  |  | 09021 | making a gift | 50-60 | 1 | 23 | 1 | 23.0 |
|  |  | 09055 | talking (sitting) | 50-60 | 3 | 351 | 9 | 39.0 |
|  |  |  |  | 60-70 | 1 | 54 | 2 | 27.0 |
|  |  | 09056 | drinking coffee & talking | 50-60 | 1 | 12 | 1 | 12.0 |
|  |  | 09057 | meeting friends | 50-60 | 1 | 27 | 1 | 27.0 |
|  |  | 09104 | bar | 50-60 | 1 | 2329 | 58 | 40.2 |
|  |  |  |  | 60-70 | 1 | 657 | 10 | 65.7 |
|  |  |  |  | 70-80 | 1 | 147 | 3 | 49.0 |
|  |  | 09107 | sightseeing | 50-60 | 1 | 372 | 11 | 33.8 |
|  |  |  |  | 60-70 | 1 | 21 | 1 | 21.0 |
|  |  | 09108 | christmas market | 50-60 | 2 | 253 | 5 | 50.6 |
|  |  | 09125 | city | 50-60 | 1 | 31 | 1 | 31.0 |
|  |  | 09130 | celebrating | 50-60 | 6 | 4165 | 99 | 42.1 |
|  |  |  |  | 60-70 | 3 | 864 | 25 | 34.6 |
|  |  |  |  | 70-80 | 2 | 128 | 3 | 42.7 |
|  |  | 09131 | halloween party | 50-60 | 1 | 58 | 2 | 29.0 |
|  |  | 09133 | celebrating birthday | 50-60 | 1 | 1121 | 16 | 70.1 |
|  |  |  |  | 60-70 | 1 | 312 | 8 | 39.0 |
|  |  | 15700 | trampoline park | 50-60 | 1 | 2614 | 60 | 43.6 |
|  |  |  |  | 60-70 | 1 | 1000 | 27 | 37.0 |
|  |  |  |  | 70-80 | 1 | 115 | 4 | 28.8 |
|  |  |  |  | 80-90 | 1 | 22 | 1 | 22.0 |
|  |  |  |  |  |  |  |  |  |
|  | Music Playing | | | | | | | |
|  |  | 10070 | playing the piano | 50-60 | 1 | 466 | 11 | 42.4 |
|  |  |  |  | 60-70 | 1 | 246 | 6 | 41.0 |
|  |  |  |  | 70-80 | 1 | 11 | 1 | 11.0 |
|  |  | 10140 | choir | 50-60 | 1 | 197 | 4 | 49.3 |
|  |  |  |  | 60-70 | 1 | 21 | 1 | 21.0 |
|  |  |  |  |  |  |  |  |  |
|  | Self Care | | | | | | | |
|  |  | 13001 | getting ready | 50-60 | 4 | 614 | 19 | 32.3 |
|  |  | 13002 | morning routine | 50-60 | 1 | 47 | 2 | 23.5 |
|  |  | 13020 | dressing | 50-60 | 2 | 298 | 4 | 74.5 |
|  |  | 13020; 13045 | dressing; hairstyling | 50-60 | 1 | 24 | 1 | 24.0 |
|  |  | 13030 | eating | 50-60 | 5 | 996 | 29 | 34.3 |
|  |  | 13030; 15745 | eating; break | 50-60 | 1 | 199 | 7 | 28.4 |
|  |  |  |  | 60-70 | 1 | 259 | 4 | 64.8 |
|  |  |  |  | 70-80 | 1 | 19 | 1 | 19.0 |
|  |  | 13040; 13042; 13045 | brushing teeth; putting on make-up; hairstyling | 50-60 | 1 | 345 | 6 | 57.5 |
|  |  |  |  | 60-70 | 1 | 44 | 1 | 44.0 |
|  |  | 13050 | showering | 50-60 | 6 | 1994 | 38 | 52.5 |
|  |  |  |  | 60-70 | 3 | 329 | 8 | 41.1 |
|  |  |  |  |  |  |  |  |  |
|  | Sports | | | | | | | |
|  |  | 15140 | competition (trainer) | 50-60 | 1 | 169 | 6 | 28.2 |
|  |  | 15141 | coaching | 50-60 | 1 | 40 | 2 | 20.0 |
|  |  |  |  | 60-70 | 1 | 3 | 1 | 3.0 |
|  |  |  |  |  |  |  |  |  |
| Occupation/ Study | Occupation/Student activities | | | | | | | |
|  |  | 09060 | sitting studying | 50-60 | 1 | 227 | 5 | 45.4 |
|  |  |  |  | 60-70 | 1 | 54 | 1 | 54.0 |
|  |  | 09062 | university preparation/follow up | 50-60 | 1 | 578 | 11 | 52.6 |
|  |  |  |  | 60-70 | 1 | 434 | 6 | 72.3 |
|  |  |  |  | 70-80 | 1 | 275 | 3 | 91.7 |
|  |  | 09062; 09063 | university preparation/follow up; group work | 50-60 | 1 | 692 | 17 | 40.7 |
|  |  | 09065 | sitting in lecture/tutorial/seminar/university | 50-60 | 5 | 1038 | 20 | 51.9 |
|  |  |  |  | 60-70 | 1 | 48 | 2 | 24.0 |
|  |  | 09066 | preparing presentation | 50-60 | 1 | 273 | 3 | 91.0 |
|  |  | 09068 | university library/copying | 50-60 | 1 | 87 | 3 | 29.0 |
|  |  |  |  | 60-70 | 1 | 15 | 1 | 15.0 |
|  |  | 11414 | working (restaurant) | 50-60 | 1 | 112 | 4 | 28.0 |
|  |  |  |  |  |  |  |  |  |
|  | Volunteer Activities | | | | | | | |
|  |  | 21080 | children sports school work shadowing | 50-60 | 2 | 518 | 13 | 39.9 |
|  |  |  |  | 60-70 | 1 | 50 | 2 | 25.0 |
|  |  |  |  |  |  |  |  |  |
| Transportation |  | | | | | | | |
|  |  |  |  |  |  |  |  |  |
|  |  | 09071; 17161 | standing in bus/tram; walking from home/work to transportation and inversely | 50-60 | 1 | 45 | 1 | 45.0 |
|  |  | 16016; 17161 | riding in a bus or train; walking from home/work to transportation and inversely | 50-60 | 2 | 221 | 5 | 44.2 |
|  |  |  |  | 60-70 | 1 | 100 | 1 | 100.0 |
|  |  |  |  | 70-80 | 1 | 5 | 1 | 5.0 |
|  |  | 16017; 16060 | riding in a tram; walking | 50-60 | 1 | 289 | 3 | 96.3 |
|  |  | 16017; 17161 | riding in a tram; walking from home/work to transportation and inversely | 50-60 | 1 | 458 | 8 | 57.3 |
|  |  |  |  | 60-70 | 1 | 171 | 4 | 42.8 |
|  |  | 16070 | going to places & way home | 50-60 | 2 | 436 | 13 | 33.5 |
|  |  |  |  |  |  |  |  |  |
|  | Bicycling | | | | | | | |
|  |  | 01015 | bicycling | 50-60 | 2 | 801 | 22 | 36.4 |
|  |  |  |  | 60-70 | 1 | 174 | 4 | 43.5 |
|  |  |  |  | 70-80 | 1 | 7 | 1 | 7.0 |
|  |  |  |  |  |  |  |  |  |
|  | Transportation | | | | | | | |
|  |  | 09071 | standing in bus/tram | 50-60 | 1 | 15 | 1 | 15.0 |
|  |  | 16010 | driving a car | 50-60 | 5 | 839 | 23 | 36.5 |
|  |  |  |  | 60-70 | 1 | 404 | 8 | 50.5 |
|  |  |  |  | 70-80 | 1 | 309 | 6 | 51.5 |
|  |  | 16015 | riding in a car | 50-60 | 1 | 22 | 2 | 11.0 |
|  |  | 16016 | riding in a bus or train | 50-60 | 4 | 1307 | 30 | 43.6 |
|  |  |  |  | 60-70 | 2 | 62 | 3 | 20.7 |
|  |  | 16017 | riding in a tram | 50-60 | 4 | 673 | 15 | 44.9 |
|  |  |  |  | 60-70 | 1 | 27 | 1 | 27.0 |
|  |  | 16016; 16017 | riding in a bus or train; riding in a tram | 50-60 | 1 | 38 | 2 | 19.0 |
|  |  | 16016; 16017; 16010 | riding in a bus or train; riding in a tram; driving a car | 50-60 | 1 | 951 | 24 | 39.6 |
|  |  |  |  | 60-70 | 1 | 222 | 4 | 55.5 |
|  |  | 16075 | outward journey/journey home | 50-60 | 2 | 2490 | 40 | 62.3 |
|  |  |  |  | 60-70 | 2 | 504 | 9 | 56.0 |
|  |  |  |  | 70-80 | 1 | 132 | 2 | 66.0 |
|  |  |  |  |  |  |  |  |  |
|  | Walking | | | | | | | |
|  |  | 16060 | walking | 50-60 | 9 | 5414 | 101 | 53.6 |
|  |  |  |  | 60-70 | 5 | 300 | 15 | 20.0 |
|  |  | 16061 | changing building | 50-60 | 1 | 63 | 2 | 31.5 |
|  |  | 17023 | walking with baggage | 50-60 | 1 | 3 | 1 | 3.0 |
|  |  | 17135 | stair climbing | 50-60 | 2 | 416 | 6 | 69.3 |
|  |  |  |  | 60-70 | 1 | 13 | 1 | 13.0 |
|  |  | 17150 | walking in the household | 50-60 | 1 | 42 | 3 | 14.0 |
|  |  |  |  | 60-70 | 1 | 73 | 2 | 36.5 |
|  |  | 17161 | walking from home/work to transportation and inversely | 50-60 | 7 | 1149 | 26 | 44.2 |
|  |  |  |  | 60-70 | 3 | 52 | 3 | 17.3 |
|  |  | 17165 | walking the dog | 50-60 | 1 | 160 | 5 | 32.0 |
|  |  |  |  |  |  |  |  |  |
| Others | Home Activities; Walking | | | | | | | |
|  |  | 05060; 16060 | food shopping; walking | 50-60 | 1 | 893 | 10 | 89.3 |
|  |  |  |  | 60-70 | 1 | 34 | 2 | 17.0 |
|  |  |  |  |  |  |  |  |  |
|  | Inactivity quiet/light; Self Care | | | | | | | |
|  |  | 07009; 13001 | getting up; getting ready | 50-60 | 1 | 564 | 10 | 56.4 |
|  |  |  |  | 60-70 | 1 | 27 | 1 | 27.0 |
|  |  | 07021; 13020 | sitting; dressing | 50-60 | 1 | 46 | 3 | 15.3 |
|  |  |  |  |  |  |  |  |  |
|  | Inactivity quiet/light; Self Care; Walking | | | | | | | |
|  |  | 07021; 13020; 16060 | sitting; dressing; walking | 50-60 | 1 | 8 | 1 | 8.0 |
|  |  |  |  |  |  |  |  |  |
|  | Self Care; Walking | | | | | | | |
|  |  | 13020; 17161 | dressing; walking from home/work to transportation and inversely | 50-60 | 1 | 327 | 8 | 40.9 |
|  |  |  |  | 60-70 | 1 | 469 | 10 | 46.9 |
|  |  |  |  | 70-80 | 1 | 73 | 4 | 18.3 |
|  |  | 13030; 16060 | eating; walking | 50-60 | 1 | 408 | 7 | 58.3 |
|  |  |  |  | 60-70 | 1 | 7 | 1 | 7.0 |
|  |  |  |  |  |  |  |  |  |
| Not Specified | | | | | | | | |
|  |  |  |  | 50-60 | 1 | 1934 | 43 | 45.0 |
|  |  |  |  | 60-70 | 1 | 45 | 2 | 22.5 |
|  |  |  |  |  |  |  |  |  |
| **Structured PA** | | | | | | | | |
| Sport | Conditioning Exercise | | | | | | | |
|  |  | 02056 | strength exercises in seminar | 50-60 | 1 | 12 | 1 | 12.0 |
|  |  | 02058 | resistance training | 50-60 | 1 | 339 | 10 | 33.9 |
|  |  | 02060 | health club exercise | 50-60 | 1 | 1150 | 28 | 41.1 |
|  |  |  |  | 60-70 | 1 | 36 | 2 | 18.0 |
|  |  | 02064 | workout | 50-60 | 1 | 193 | 5 | 38.6 |
|  |  |  |  | 60-70 | 1 | 29 | 2 | 14.5 |
|  |  | 15740 | exercise/sports/unisport | 50-60 | 2 | 1794 | 48 | 37.4 |
|  |  |  |  | 60-70 | 2 | 530 | 12 | 44.2 |
|  |  |  |  | 70-80 | 1 | 1380 | 18 | 76.7 |
|  |  |  |  | 80-90 | 1 | 338 | 14 | 24.1 |
|  |  |  |  | 90-100 | 1 | 27 | 1 | 27.0 |
|  |  |  |  |  |  |  |  |  |
|  | Dancing | | | | | | | |
|  |  | 03031 | dancing | 50-60 | 1 | 99 | 5 | 19.8 |
|  |  |  |  |  |  |  |  |  |
|  | Running | | | | | | | |
|  |  | 12210; 02058 | running training; resistance training | 50-60 | 1 | 1205 | 35 | 34.4 |
|  |  |  |  | 60-70 | 1 | 128 | 3 | 42.7 |
|  |  | 12020 | jogging | 50-60 | 1 | 902 | 21 | 43.0 |
|  |  |  |  | 60-70 | 1 | 1459 | 28 | 52.1 |
|  |  |  |  | 70-80 | 1 | 1646 | 20 | 82.3 |
|  |  |  |  | 80-90 | 1 | 1059 | 7 | 151.3 |
|  |  |  |  | 90-100 | 1 | 68 | 3 | 22.7 |
|  |  | 12220 | running circuit | 50-60 | 1 | 992 | 22 | 45.1 |
|  |  |  |  | 60-70 | 1 | 948 | 20 | 47.4 |
|  |  |  |  | 70-80 | 1 | 649 | 13 | 49.9 |
|  |  |  |  | 80-90 | 1 | 41 | 2 | 20.5 |
|  |  |  |  |  |  |  |  |  |
|  | Sports | | | | | | | |
|  |  | 15300 | handstand training | 50-60 | 1 | 144 | 3 | 48.0 |
|  |  |  |  | 60-70 | 1 | 56 | 2 | 28.0 |
|  |  |  |  | 70-80 | 1 | 24 | 1 | 24.0 |
|  |  | 15320 | handball training | 50-60 | 2 | 2123 | 56 | 37.9 |
|  |  |  |  | 60-70 | 2 | 1844 | 48 | 38.4 |
|  |  |  |  | 70-80 | 2 | 1249 | 29 | 43.1 |
|  |  |  |  | 80-90 | 1 | 160 | 6 | 26.7 |
|  |  | 15610 | soccer training | 50-60 | 1 | 3925 | 88 | 44.6 |
|  |  |  |  | 60-70 | 1 | 1127 | 30 | 37.6 |
|  |  |  |  | 70-80 | 1 | 93 | 4 | 23.3 |
|  |  |  |  |  |  |  |  |  |
|  | Walking | | | | | | | |
|  |  | 17315 | nordic walking | 50-60 | 1 | 1341 | 20 | 67.1 |
|  |  |  |  | 60-70 | 1 | 566 | 7 | 80.9 |
|  |  |  |  | 70-80 | 1 | 24 | 1 | 24.0 |
|  |  |  |  |  |  |  |  |  |
|  | Water Activities | | | | | | | |
|  |  | 18251 | swimming | 50-60 | 1 | 355 | 9 | 39.4 |
|  |  |  |  | 60-70 | 1 | 35 | 1 | 35.0 |

Table 2: Description of the specific type of incidental lifestyle PA and the specific type of structured PA of n=12 office workers in the different heart rate reserve (HRR) zones, the number of subjects per activity and their total duration during waking time, their frequency, and their mean duration of activity when it occurred (total duration of waking time in HRR zones ≥ 50% = 192965 s (53.60 h); total frequency in waking time in HRR zones ≥ 50% = 4161).

| **Domain** | **Major heading of**  **compendium** | **Code** | **Type of activity** | **Zone of HRR %** | **Number of participants mentioning the activity** | **Total duration in activity during waking time [s]** | **Frequency of activity in waking time** | **Mean duration of each activity [s]** |
| --- | --- | --- | --- | --- | --- | --- | --- | --- |
| **Incidental Lifestyle PA** | | | | | | | | |
| Household | Home Activities | | | | | | | |
|  |  | 05030 | dishwasher | 50-60 | 1 | 121 | 3 | 40.3 |
|  |  | 05030; 05050 | dishwasher; cooking/food preparation | 50-60 | 1 | 17 | 1 | 17.0 |
|  |  | 05030; 05068 | dishwasher; making a coffee/tea | 50-60 | 1 | 149 | 3 | 49.7 |
|  |  | 05031 | cleaning | 50-60 | 5 | 3060 | 66 | 46.4 |
|  |  |  |  | 60-70 | 5 | 249 | 12 | 20.8 |
|  |  |  |  | 70-80 | 1 | 12 | 1 | 12.0 |
|  |  | 05031; 05035 | cleaning; kitchen activity | 50-60 | 1 | 26 | 1 | 26.0 |
|  |  | 05031; 05169 | cleaning; playing/romping with children | 50-60 | 1 | 1073 | 14 | 76.6 |
|  |  |  |  | 60-70 | 1 | 131 | 4 | 32.8 |
|  |  | 05033; 05043 | cleaning apartment; vacuuming | 50-60 | 1 | 286 | 9 | 31.8 |
|  |  |  |  | 60-70 | 1 | 33 | 1 | 33.0 |
|  |  | 05035 | kitchen activity | 50-60 | 4 | 1666 | 38 | 43.8 |
|  |  |  |  | 60-70 | 1 | 172 | 4 | 43.0 |
|  |  | 05035; 05050 | kitchen activity; cooking/food preparation | 50-60 | 1 | 863 | 8 | 107.9 |
|  |  |  |  | 60-70 | 1 | 67 | 2 | 33.5 |
|  |  | 05040 | carrying out trash | 50-60 | 1 | 413 | 6 | 68.8 |
|  |  | 05041 | wash dishes | 50-60 | 1 | 90 | 1 | 90.0 |
|  |  | 05043 | vacuuming | 50-60 | 1 | 1093 | 16 | 68.3 |
|  |  |  |  | 60-70 | 1 | 314 | 8 | 39.3 |
|  |  | 05050 | cooking/food preparation | 50-60 | 5 | 8103 | 141 | 57.5 |
|  |  |  |  | 60-70 | 4 | 664 | 26 | 25.5 |
|  |  | 05050; 05068 | cooking/food preparation; making a coffee/tea | 50-60 | 1 | 15 | 1 | 15.0 |
|  |  | 05053 | feeding cat | 50-60 | 1 | 9 | 1 | 9.0 |
|  |  | 05060 | food shopping | 50-60 | 9 | 1769 | 35 | 50.5 |
|  |  |  |  | 60-70 | 1 | 26 | 1 | 26.0 |
|  |  | 05061 | material handling | 50-60 | 1 | 50 | 2 | 25.0 |
|  |  | 05067 | cracking nuts | 50-60 | 1 | 266 | 6 | 44.3 |
|  |  | 05068; 05136 | making a coffee/tea; cleaning washbasin | 50-60 | 1 | 75 | 3 | 25.0 |
|  |  | 05070 | ironing | 50-60 | 1 | 1999 | 29 | 68.9 |
|  |  |  |  | 60-70 | 1 | 397 | 9 | 44.1 |
|  |  | 05070; 05135 | ironing; cleaning bathroom | 50-60 | 1 | 241 | 10 | 24.1 |
|  |  |  |  | 60-70 | 1 | 60 | 2 | 30.0 |
|  |  | 05089 | laundry | 50-60 | 1 | 952 | 26 | 36.6 |
|  |  |  |  | 60-70 | 1 | 168 | 5 | 33.6 |
|  |  | 05091 | hanging laundry | 50-60 | 4 | 330 | 12 | 27.5 |
|  |  |  |  | 60-70 | 2 | 47 | 2 | 23.5 |
|  |  | 05091; 05094 | hanging laundry; take off laundry | 50-60 | 1 | 146 | 6 | 24.3 |
|  |  | 05094 | take off laundry | 50-60 | 1 | 25 | 1 | 25.0 |
|  |  | 05095 | putting away clothes | 50-60 | 1 | 47 | 3 | 15.7 |
|  |  | 05097 | folding laundry | 50-60 | 1 | 75 | 1 | 75.0 |
|  |  | 05100 | making bed | 50-60 | 1 | 26 | 2 | 13.0 |
|  |  |  |  | 60-70 | 1 | 15 | 1 | 15.0 |
|  |  | 05135 | cleaning bathroom | 50-60 | 1 | 1227 | 35 | 35.1 |
|  |  |  |  | 60-70 | 1 | 14 | 1 | 14.0 |
|  |  | 05168 | take/get child to/from kindergarten | 50-60 | 1 | 388 | 13 | 29.9 |
|  |  |  |  | 60-70 | 1 | 35 | 1 | 35.0 |
|  |  | 05169 | playing/romping with children | 50-60 | 2 | 363 | 10 | 36.3 |
|  |  |  |  | 60-70 | 1 | 38 | 1 | 38.0 |
|  |  | 05184 | getting child ready | 50-60 | 1 | 371 | 9 | 41.2 |
|  |  |  |  | 60-70 | 1 | 1 | 1 | 1.0 |
|  |  | 05187 | putting child to bed | 50-60 | 1 | 9 | 1 | 9.0 |
|  |  | 05210 | housework | 50-60 | 5 | 4289 | 99 | 43.3 |
|  |  |  |  | 60-70 | 3 | 999 | 29 | 34.5 |
|  |  |  |  | 70-80 | 1 | 200 | 5 | 40.0 |
|  |  | 05211 | housework (cooking. cleaning) | 50-60 | 1 | 185 | 6 | 30.8 |
|  |  |  |  | 60-70 | 1 | 15 | 1 | 15.0 |
|  |  | 05215 | cleaning terrace | 50-60 | 1 | 10 | 1 | 10.0 |
|  |  | 05230 | clean up campsite | 50-60 | 1 | 1638 | 23 | 71.2 |
|  |  |  |  | 60-70 | 1 | 1049 | 16 | 65.6 |
|  |  |  |  | 70-80 | 1 | 46 | 2 | 23.0 |
|  |  |  |  |  |  |  |  |  |
|  | Home Repair | | | | | | | |
|  |  | 06245 | working on roof | 50-60 | 1 | 2155 | 37 | 58.2 |
|  |  |  |  | 60-70 | 1 | 222 | 7 | 31.7 |
|  |  |  |  |  |  |  |  |  |
|  | Lawn and Garden | | | | | | | |
|  |  | 08000 | working physically outside | 50-60 | 1 | 377 | 8 | 47.1 |
|  |  |  |  | 60-70 | 1 | 34 | 2 | 17.0 |
|  |  | 08263 | clean up garden | 50-60 | 1 | 150 | 6 | 25.0 |
|  |  |  |  |  |  |  |  |  |
| Leisure Time | Inactivity quiet/light | | | | | | | |
|  |  | 07009 | getting up | 50-60 | 3 | 462 | 8 | 57.8 |
|  |  |  |  | 60-70 | 2 | 412 | 8 | 51.5 |
|  |  |  |  | 70-80 | 1 | 83 | 5 | 16.6 |
|  |  |  |  | 80-90 | 1 | 68 | 1 | 68.0 |
|  |  | 07010 | watching television (lying) | 50-60 | 2 | 322 | 5 | 64.4 |
|  |  | 07011; 07021 | lying; sitting | 50-60 | 1 | 45 | 2 | 22.5 |
|  |  |  |  | 60-70 | 1 | 78 | 1 | 78.0 |
|  |  | 07012 | lying on bed/couch | 50-60 | 3 | 296 | 13 | 22.8 |
|  |  | 07015 | watching television. series. movies. Netflix. Youtube | 50-60 | 5 | 556 | 20 | 27.8 |
|  |  |  |  | 60-70 | 2 | 23 | 2 | 11.5 |
|  |  | 07020 | watching television (sitting) | 50-60 | 1 | 254 | 9 | 28.2 |
|  |  | 07021 | sitting | 50-60 | 8 | 3745 | 84 | 44.6 |
|  |  |  |  | 60-70 | 4 | 188 | 11 | 17.1 |
|  |  |  |  | 70-80 | 3 | 155 | 5 | 31.0 |
|  |  |  |  | 80-90 | 1 | 40 | 1 | 40.0 |
|  |  | 07021; 07040 | sitting; standing | 50-60 | 1 | 41 | 1 | 41.0 |
|  |  | 07040 | standing | 50-60 | 5 | 1939 | 37 | 52.4 |
|  |  |  |  | 60-70 | 1 | 148 | 5 | 29.6 |
|  |  | 07070 | reclining. reading | 50-60 | 1 | 41 | 1 | 41.0 |
|  |  | 07071 | reading | 50-60 | 2 | 801 | 17 | 47.1 |
|  |  |  |  | 60-70 | 1 | 13 | 1 | 13.0 |
|  |  | 07085 | break after sport | 50-60 | 1 | 45 | 2 | 22.5 |
|  |  |  |  | 60-70 | 1 | 27 | 2 | 13.5 |
|  |  |  |  | 70-80 | 1 | 254 | 2 | 127.0 |
|  |  |  |  |  |  |  |  |  |
|  | Miscellaneous | | | | | | | |
|  |  | 09050 | telephone call (standing) | 50-60 | 1 | 16 | 1 | 16.0 |
|  |  | 09052 | telephone call (sitting) | 50-60 | 1 | 9 | 1 | 9.0 |
|  |  | 09053 | telephone call/conversation | 50-60 | 1 | 87 | 4 | 21.8 |
|  |  | 09059 | conversation (sitting) | 50-60 | 1 | 63 | 4 | 15.8 |
|  |  |  |  | 60-70 | 1 | 24 | 1 | 24.0 |
|  |  | 09124 | ice cream parlour | 50-60 | 1 | 42 | 1 | 42.0 |
|  |  | 09140 | parents' evening | 50-60 | 1 | 81 | 3 | 27.0 |
|  |  | 09210 | crafting | 50-60 | 1 | 47 | 3 | 15.7 |
|  |  | 09220 | shopping | 50-60 | 1 | 458 | 6 | 76.3 |
|  |  |  |  | 60-70 | 1 | 64 | 2 | 32.0 |
|  |  | 09250 | home | 50-60 | 1 | 5 | 1 | 5.0 |
|  |  |  |  |  |  |  |  |  |
|  | Self Care | | | | | | | |
|  |  | 13008; 13040 | morning toilet/brushing teeth | 50-60 | 1 | 239 | 8 | 29.9 |
|  |  |  |  | 60-70 | 1 | 92 | 2 | 46.0 |
|  |  | 13009 | WC | 50-60 | 1 | 160 | 4 | 40.0 |
|  |  | 13009; 13043 | WC; bathroom | 50-60 | 1 | 1135 | 17 | 66.8 |
|  |  |  |  | 60-70 | 1 | 135 | 3 | 45.0 |
|  |  | 13010 | bathing | 50-60 | 1 | 172 | 2 | 86.0 |
|  |  | 13010; 13041; 13045 | bathing; skin care; hairstyling | 50-60 | 1 | 627 | 15 | 41.8 |
|  |  |  |  | 60-70 | 1 | 22 | 1 | 22.0 |
|  |  | 13020 | dressing | 50-60 | 5 | 1475 | 44 | 33.5 |
|  |  |  |  | 60-70 | 4 | 564 | 17 | 33.2 |
|  |  |  |  | 70-80 | 3 | 118 | 5 | 23.6 |
|  |  |  |  | 80-90 | 1 | 93 | 2 | 46.5 |
|  |  |  |  | 90-100 | 1 | 139 | 1 | 139.0 |
|  |  | 13020; 13040 | dressing; brushing teeth | 50-60 | 1 | 258 | 11 | 23.5 |
|  |  | 13020; 13050 | dressing; showering | 50-60 | 1 | 273 | 8 | 34.1 |
|  |  |  |  | 60-70 | 1 | 204 | 5 | 40.8 |
|  |  | 13030 | eating | 50-60 | 10 | 4241 | 101 | 42.0 |
|  |  |  |  | 60-70 | 4 | 949 | 25 | 38.0 |
|  |  |  |  | 70-80 | 1 | 766 | 20 | 38.3 |
|  |  |  |  | 80-90 | 1 | 348 | 8 | 43.5 |
|  |  |  |  | 90-100 | 1 | 16 | 1 | 16.0 |
|  |  | 13031 | drinking coffee/tea | 50-60 | 1 | 72 | 1 | 72.0 |
|  |  | 13040; 13051 | brushing teeth; washing | 50-60 | 1 | 18 | 1 | 18.0 |
|  |  | 13043 | bathroom | 50-60 | 7 | 2623 | 79 | 33.2 |
|  |  |  |  | 60-70 | 3 | 318 | 13 | 24.5 |
|  |  |  |  | 70-80 | 1 | 59 | 3 | 19.7 |
|  |  |  |  | 80-90 | 1 | 78 | 2 | 39.0 |
|  |  |  |  | 90-100 | 1 | 33 | 1 | 33.0 |
|  |  | 13043; 13050 | bathroom; showering | 50-60 | 2 | 486 | 8 | 60.8 |
|  |  | 13044 | washing hair | 50-60 | 1 | 720 | 8 | 90.0 |
|  |  |  |  | 60-70 | 1 | 42 | 1 | 42.0 |
|  |  | 13045 | hairstyling | 50-60 | 2 | 570 | 12 | 47.5 |
|  |  |  |  | 60-70 | 2 | 90 | 3 | 30.0 |
|  |  | 13050 | showering | 50-60 | 5 | 3913 | 76 | 51.5 |
|  |  |  |  | 60-70 | 4 | 1983 | 47 | 42.2 |
|  |  |  |  | 70-80 | 3 | 453 | 12 | 37.8 |
|  |  |  |  |  |  |  |  |  |
| Occupation/ Study | Occupation/Student activities | | | | | | | |
|  |  | 09015; 11594 | copying/scanning; office_telephone call (sitting) | 50-60 | 1 | 194 | 10 | 19.4 |
|  |  |  |  | 60-70 | 1 | 70 | 3 | 23.3 |
|  |  | 11572 | tea/coffee break | 50-60 | 1 | 316 | 4 | 79.0 |
|  |  | 11577 | emergency call processing | 50-60 | 1 | 3 | 1 | 3.0 |
|  |  | 11578 | home office (sitting) | 50-60 | 1 | 648 | 10 | 64.8 |
|  |  | 11581 | office work (sitting) | 50-60 | 6 | 3606 | 74 | 48.7 |
|  |  |  |  | 60-70 | 3 | 183 | 7 | 26.1 |
|  |  |  |  | 70-80 | 1 | 45 | 1 | 45.0 |
|  |  | 11582 | office work (sitting /walking briefly) | 50-60 | 2 | 238 | 12 | 19.8 |
|  |  |  |  | 60-70 | 1 | 25 | 1 | 25.0 |
|  |  | 11585 | meeting | 50-60 | 2 | 370 | 11 | 33.6 |
|  |  |  |  | 60-70 | 1 | 12 | 1 | 12.0 |
|  |  | 11586 | edit emails | 50-60 | 1 | 60 | 2 | 30.0 |
|  |  | 11588 | cleaning up office/organization | 50-60 | 1 | 227 | 11 | 20.6 |
|  |  |  |  | 60-70 | 1 | 84 | 4 | 21.0 |
|  |  | 11591 | office_conversation (sitting) | 50-60 | 1 | 143 | 5 | 28.6 |
|  |  |  | office_conversation (sitting. difficult) | 50-60 | 1 | 26 | 3 | 8.7 |
|  |  |  |  | 60-70 | 1 | 314 | 4 | 78.5 |
|  |  |  |  | 70-80 | 1 | 34 | 2 | 17.0 |
|  |  | 11592 | office_conversation (standing. difficult) | 50-60 | 1 | 105 | 2 | 52.5 |
|  |  |  |  | 60-70 | 1 | 32 | 1 | 32.0 |
|  |  | 11592; 11595 | office_conversation (standing); telephone call (standing) | 50-60 | 1 | 338 | 10 | 33.8 |
|  |  | 11594 | office_telephone call (sitting) | 50-60 | 1 | 337 | 15 | 22.5 |
|  |  |  |  | 60-70 | 1 | 117 | 4 | 29.3 |
|  |  | 11770 | working on the PC | 50-60 | 1 | 915 | 32 | 28.6 |
|  |  |  |  | 60-70 | 1 | 79 | 5 | 15.8 |
|  |  | 11791 | walking in the office | 50-60 | 1 | 309 | 5 | 61.8 |
|  |  |  |  |  |  |  |  |  |
| Transportation |  | | | | | | | |
|  |  | 16010; 17161 | driving a car; walking from home/work to transportation and inversely | 50-60 | 1 | 453 | 2 | 226.5 |
|  |  |  |  | 60-70 | 1 | 155 | 2 | 77.5 |
|  |  | 16010; 17135; 17161 | driving a car; stair climbing; walking from home/work to transportation and inversely | 50-60 | 1 | 368 | 11 | 33.5 |
|  |  |  |  | 60-70 | 1 | 158 | 7 | 22.6 |
|  |  |  |  | 70-80 | 1 | 33 | 1 | 33.0 |
|  |  |  |  |  |  |  |  |  |
|  | Bicycling | | | | | | | |
|  |  | 01015 | bicycling | 50-60 | 2 | 2783 | 74 | 37.6 |
|  |  |  |  | 60-70 | 2 | 2132 | 55 | 38.8 |
|  |  |  |  | 70-80 | 2 | 827 | 22 | 37.6 |
|  |  |  |  | 80-90 | 1 | 130 | 3 | 43.3 |
|  |  |  |  |  |  |  |  |  |
|  | Transportation | | | | | | | |
|  |  | 16010 | driving a car | 50-60 | 11 | 7924 | 180 | 44.0 |
|  |  |  |  | 60-70 | 8 | 948 | 29 | 32.7 |
|  |  | 16010; 16012 | driving a car; refuelling | 50-60 | 1 | 89 | 1 | 89.0 |
|  |  | 16015 | riding in a car | 50-60 | 2 | 199 | 6 | 33.2 |
|  |  | 16030 | motorcycle | 50-60 | 1 | 645 | 20 | 32.3 |
|  |  |  |  | 60-70 | 1 | 259 | 5 | 51.8 |
|  |  |  |  |  |  |  |  |  |
|  | Walking | | | | | | | |
|  |  | 16060 | walking | 50-60 | 10 | 6371 | 155 | 41.1 |
|  |  |  |  | 60-70 | 4 | 720 | 20 | 36.0 |
|  |  | 16065 | walking (*jogging*) | 50-60 | 1 | 1616 | 15 | 107.7 |
|  |  |  |  | 60-70 | 1 | 462 | 7 | 66.0 |
|  |  |  |  | 70-80 | 1 | 79 | 3 | 26.3 |
|  |  |  |  | 80-90 | 1 | 23 | 2 | 11.5 |
|  |  |  |  | 90-100 | 1 | 187 | 1 | 187.0 |
|  |  | 17025 | stair climbing with baggage | 50-60 | 1 | 37 | 2 | 18.5 |
|  |  | 17135 | stair climbing | 50-60 | 12 | 12893 | 327 | 39.4 |
|  |  |  |  | 60-70 | 9 | 2740 | 95 | 28.8 |
|  |  |  |  | 70-80 | 3 | 355 | 15 | 23.7 |
|  |  |  |  | 80-90 | 1 | 61 | 2 | 30.5 |
|  |  | 17136 | going downstairs | 50-60 | 10 | 1548 | 40 | 38.7 |
|  |  |  |  | 60-70 | 4 | 173 | 4 | 43.3 |
|  |  |  |  | 70-80 | 1 | 44 | 2 | 22.0 |
|  |  | 17150 | walking in the household | 50-60 | 5 | 2257 | 51 | 44.3 |
|  |  |  |  | 60-70 | 1 | 169 | 3 | 56.3 |
|  |  | 17155 | strolling | 50-60 | 2 | 1069 | 19 | 56.3 |
|  |  |  |  | 60-70 | 1 | 30 | 1 | 30.0 |
|  |  | 17161 | walking from home/work to transportation and inversely | 50-60 | 8 | 4379 | 106 | 41.3 |
|  |  |  |  | 60-70 | 3 | 915 | 24 | 38.1 |
|  |  |  |  | 70-80 | 2 | 76 | 3 | 25.3 |
|  |  |  |  | 80-90 | 1 | 19 | 1 | 19.0 |
|  |  | 17165 | walking the dog | 50-60 | 1 | 3575 | 36 | 99.3 |
|  |  |  |  | 60-70 | 1 | 175 | 6 | 29.2 |
|  |  |  |  | 70-80 | 1 | 48 | 4 | 12.0 |
|  |  |  |  | 80-90 | 1 | 492 | 4 | 123.0 |
|  |  |  |  | 90-100 | 1 | 12 | 1 | 12.0 |
|  |  |  |  |  |  |  |  |  |
| Others | Conditioning Exercise; Self Care | | | | | | | |
|  |  | 02101; 13050 | stretching; showering | 50-60 | 1 | 101 | 5 | 20.2 |
|  |  |  |  |  |  |  |  |  |
|  | Home Activities; Walking | | | | | | | |
|  |  | 05031; 17150 | cleaning; walking in the household | 50-60 | 1 | 845 | 9 | 93.9 |
|  |  |  |  | 60-70 | 1 | 74 | 4 | 18.5 |
|  |  | 05034; 16060 | cleaning refrigerator; walking | 50-60 | 1 | 153 | 4 | 38.3 |
|  |  |  |  | 60-70 | 1 | 11 | 1 | 11.0 |
|  |  | 05035; 17136 | kitchen activity; going downstairs | 70-80 | 1 | 564 | 1 | 564.0 |
|  |  | 05035; 17150 | kitchen activity; walking in the household | 50-60 | 1 | 202 | 1 | 202.0 |
|  |  | 05059; 17135 | unloading groceries; stair climbing | 50-60 | 1 | 487 | 6 | 81.2 |
|  |  | 05089; 17150 | laundry; walking in the household | 50-60 | 1 | 13 | 1 | 13.0 |
|  |  | 05169; 16060 | playing/romping with children; walking | 50-60 | 1 | 630 | 13 | 48.5 |
|  |  |  |  |  |  |  |  |  |
|  | Home Activities; Self Care | | | | | | | |
|  |  | 05050; 13009; 13043 | cooking/food preparation; WC; bathroom | 50-60 | 1 | 273 | 4 | 68.3 |
|  |  |  |  | 60-70 | 1 | 12 | 1 | 12.0 |
|  |  | 05050; 13020 | cooking/food preparation; dressing | 50-60 | 1 | 368 | 9 | 40.9 |
|  |  |  |  | 60-70 | 1 | 315 | 7 | 45.0 |
|  |  |  |  | 70-80 | 1 | 8 | 1 | 8.0 |
|  |  | 05050; 13030 | cooking/food preparation; eating | 50-60 | 1 | 408 | 9 | 45.3 |
|  |  |  |  |  |  |  |  |  |
|  | Home Activities; Transportation | | | | | | | |
|  |  | 05060; 16010 | food shopping; driving a car | 50-60 | 2 | 1188 | 27 | 44.0 |
|  |  |  |  | 60-70 | 1 | 20 | 2 | 10.0 |
|  |  | 05062; 16010 | errands; driving a car | 50-60 | 1 | 27 | 1 | 27.0 |
|  |  |  |  |  |  |  |  |  |
|  | Home Activities; Transportation; Walking | | | | | | | |
|  |  | 05060; 16010; 17161 | food shopping; driving a car; walking from home/work to transportation and inversely | 50-60 | 1 | 1369 | 30 | 45.6 |
|  |  |  |  | 60-70 | 1 | 229 | 7 | 32.7 |
|  |  |  |  |  |  |  |  |  |
|  | Inactivity quiet/light; Self Care | | | | | | | |
|  |  | 07009; 13043 | getting up; bathroom | 50-60 | 1 | 39 | 1 | 39.0 |
|  |  | 07012; 13030; 13043 | lying on bed/couch; eating; bathroom | 50-60 | 1 | 76 | 5 | 15.2 |
|  |  |  |  |  |  |  |  |  |
|  | Miscellaneous; Self Care | | | | | | | |
|  |  | 09030; 13031 | reading (sitting); drinking coffee/tea | 50-60 | 1 | 22 | 2 | 11.0 |
|  |  |  |  |  |  |  |  |  |
|  | Occupation/Student activities; Walking | | | | | | | |
|  |  | 11588; 16060 | cleaning up office/organization; walking | 50-60 | 1 | 211 | 4 | 52.8 |
|  |  |  |  | 60-70 | 1 | 37 | 2 | 18.5 |
|  |  |  |  | 70-80 | 1 | 10 | 1 | 10.0 |
|  |  |  |  |  |  |  |  |  |
|  | Self Care; Walking | | | | | | | |
|  |  | 13020; 17161 | dressing; walking from home/work to transportation and inversely | 50-60 | 1 | 7 | 1 | 7.0 |
|  |  | 13043; 17150 | bathroom; walking in the household | 50-60 | 1 | 54 | 2 | 27.0 |
|  |  |  |  |  |  |  |  |  |
| Not Specified | | | | | | | | |
|  |  |  |  | 50-60 | 2 | 118 | 4 | 29.5 |
|  |  |  |  |  |  |  |  |  |
| **Structured PA** | | | | | | | | |
| Sport | Bicycling | | | | | | | |
|  |  | 01009 | mountain biking | 50-60 | 1 | 1185 | 21 | 56.4 |
|  |  |  |  | 60-70 | 1 | 1696 | 43 | 39.4 |
|  |  |  |  | 70-80 | 1 | 2613 | 66 | 39.6 |
|  |  |  |  | 80-90 | 1 | 3259 | 59 | 55.2 |
|  |  |  |  | 90-100 | 1 | 1027 | 21 | 48.9 |
|  |  |  |  |  |  |  |  |  |
|  | Conditioning Exercise | | | | | | | |
|  |  | 02023 | push ups. situps. squats | 50-60 | 1 | 397 | 9 | 44.1 |
|  |  |  |  | 60-70 | 1 | 126 | 4 | 31.5 |
|  |  |  |  | 70-80 | 1 | 29 | 1 | 29.0 |
|  |  | 02025 | abdominal workout | 50-60 | 1 | 421 | 16 | 26.3 |
|  |  |  |  | 60-70 | 1 | 162 | 7 | 23.1 |
|  |  | 02025; 02069 | abdominal workout; deep musculature training | 50-60 | 1 | 110 | 4 | 27.5 |
|  |  | 02029 | back training | 50-60 | 1 | 3 | 1 | 3.0 |
|  |  | 02037 | circuit training | 50-60 | 1 | 443 | 12 | 36.9 |
|  |  |  |  | 60-70 | 1 | 1418 | 20 | 70.9 |
|  |  |  |  | 70-80 | 1 | 514 | 16 | 32.1 |
|  |  |  |  | 80-90 | 1 | 468 | 9 | 52.0 |
|  |  |  |  | 90-100 | 1 | 301 | 4 | 75.3 |
|  |  | 02049 | treadmill | 50-60 | 1 | 329 | 5 | 65.8 |
|  |  |  |  | 60-70 | 1 | 102 | 3 | 34.0 |
|  |  |  |  | 70-80 | 1 | 138 | 4 | 34.5 |
|  |  |  |  | 80-90 | 1 | 333 | 9 | 37.0 |
|  |  |  |  | 90-100 | 1 | 1970 | 8 | 246.3 |
|  |  | 02067 | functional body training | 50-60 | 1 | 388 | 9 | 43.1 |
|  |  |  |  | 60-70 | 1 | 487 | 11 | 44.3 |
|  |  |  |  | 70-80 | 1 | 25 | 2 | 12.5 |
|  |  | 15740 | sport | 50-60 | 2 | 1128 | 37 | 30.5 |
|  |  |  |  | 60-70 | 2 | 4357 | 70 | 62.2 |
|  |  |  |  | 70-80 | 2 | 10297 | 74 | 139.2 |
|  |  |  |  | 80-90 | 2 | 3759 | 58 | 64.8 |
|  |  |  |  | 90-100 | 2 | 4733 | 35 | 135.2 |
|  |  |  |  |  |  |  |  |  |
|  | Dancing | | | | | | | |
|  |  | 03023 | dance workout/dance aerobics | 50-60 | 1 | 523 | 15 | 34.9 |
|  |  |  |  | 60-70 | 1 | 1111 | 22 | 50.5 |
|  |  |  |  | 70-80 | 1 | 900 | 22 | 40.9 |
|  |  |  |  | 80-90 | 1 | 238 | 9 | 26.4 |
|  |  |  |  |  |  |  |  |  |
|  | Sports | | | | | | | |
|  |  | 15670 | qi gong (shaking exercise) | 50-60 | 1 | 11 | 2 | 5.5 |
|  |  |  |  | 60-70 | 1 | 11 | 2 | 5.5 |
|  |  |  |  | 70-80 | 1 | 20 | 2 | 10.0 |
|  |  |  |  | 80-90 | 1 | 25 | 2 | 12. |
|  |  |  |  | 90-100 | 1 | 8 | 1 | 8.0 |
